# Pairing nuts and dried fruit for cardiometabolic health

**DOI:** 10.1186/s12937-016-0142-4

**Published:** 2016-03-05

**Authors:** Arianna Carughi, Mary Jo Feeney, Penny Kris-Etherton, Victor Fulgoni, Cyril W. C. Kendall, Mònica Bulló, Densie Webb

**Affiliations:** 1 Sun-Maid Growers of California, 13525 S. Bethel Ave., Kingsburg, CA 93631 USA; 2 Consultant to the Food and Agriculture Industries, 11030 Mora Dr, Los Altos, CA 94024 USA; 3 Department of Nutritional Sciences, The Pennsylvania State University, 319 Chandlee Laboratory, University Park, PA 16802 USA; 4 Nutrition Impact, LLC, 9725 D Drive North, Battle Creek, MI 49014 USA; 5 Department of Nutrition Sciences, Faculty of Medicine, University of Toronto, 32 Ridley Gardens, Toronto, ON M6R 2T8 Canada; 6 Human Nutrition Unit, Faculty of Medicine and Health Sciences, Rovira i Virgili University, Sant Llorenç, 21, 43201 Reus, Spain; 7 Health and Nutrition Communications, 8014 Greenslope Drive, Austin, TX 78759 USA

## Abstract

Certain dietary patterns, in which fruits and nuts are featured prominently, reduce risk of diabetes and cardiovascular disease. However, estimated fruit consumption historically in the U.S. has been lower than recommendations. Dried fruit intake is even lower with only about 6.9 % of the adult population reporting any consumption. The 2015 Dietary Guidelines Advisory Committee identified a gap between recommended fruit and vegetable intakes and the amount the population consumes. Even fewer Americans consume tree nuts, which are a nutrient-dense food, rich in bioactive compounds and healthy fatty acids. Consumption of fruits and nuts has been associated with reduced risk of cardiometabolic disease. An estimated 5.5 to 8.4 % of U.S. adults consume tree nuts and/or tree nut butter. This review examines the potential of pairing nuts and dried fruit to reduce cardiometabolic risk factors and focuses on emerging data on raisins and pistachios as representative of each food category. Evidence suggests that increasing consumption of both could help improve Americans’ nutritional status and reduce the risk of chronic diseases.

## Review

### Introduction

It is well established that certain dietary patterns decrease disease risk and benefit the management of diabetes and cardiovascular disease [1–3]. A substantial body of research demonstrates that diets rich in fruits and vegetables can reduce the risk of overweight and obesity, cardiovascular disease, type 2 diabetes and hypertension [4, 5]. Fruit and vegetable intakes are, in fact, the only dietary characteristic consistently associated with every conclusion statement across health outcomes in the 2015 U.S. Dietary Guidelines Advisory Committee (DGAC) report [2]. Despite the overwhelming evidence of the health benefits associated with consuming fruits and vegetables, the DGAC identified a large gap between recommended fruit and vegetable intakes and the average amounts the population consumes [2]. While the role of dried fruit in health is less well studied, health agencies around the world recommend them as a convenient way to incorporate more fruit into the diet.

The health benefits of tree nuts in relation to cardiovascular disease risk reduction have been widely studied [5–11]. More recently, nut consumption has also been associated with a lower risk of all cause, CVD, and cancer mortality [12–14]. In addition, the consumption of certain nuts, including pistachios, has been associated with improvements in the regulation of blood glucose and insulin, markers of inflammation, endothelial function [15], and other related metabolic risk markers [5]. Nuts and dried fruits are healthful foods because of their nutrient profiles. They provide dietary fiber, potassium (K) and a variety of health protective bioactive compounds. Nuts also are a source of protein and monounsaturated (MUFA) and polyunsaturated fatty acids (PUFA).

This review examines the role of dried fruits and nuts in supporting cardiometabolic health and reducing CVD disease risk. It addresses the contribution that tree nuts and dried fruit make to U.S. nutrient intake and diet quality; the beneficial effects of tree nuts on cardiometabolic risk factors; and the effect of dried fruits on glycemia, insulinemia, and metabolic risk factors. It focuses on emerging research with raisins, the most commonly consumed dried fruit, and pistachios as representatives of each food category.

### Nutrient contribution of dried fruit and nuts and their effect on diet quality

Three healthy dietary patterns are recommended by the 2015 Dietary Guidelines for Americans—a Healthy US-style Pattern, a Healthy Vegetarian Pattern, and a Healthy Mediterranean-style pattern. Fruits, nuts, and seeds play a prominent role in all three of these food-based dietary patterns, which recommend between 2 and 2 ½ cup equivalents of fruit a day and 4-7 oz equivalents per week of nuts and seeds [16].

#### Fruit and dried fruit

Fruit consumption in the U.S. has been low historically and has changed little in recent years. Data from the Centers for Disease Control and Prevention (CDC) indicate that 39 % of adults consume fruit less than once per day [17] and in a state-by-state survey, no state met the fruit intake target [18]. Children generally fare better with fruit intake, but not by much. Data from National Health and Nutrition Examination Survey (NHANES) 2003–2010 showed that while intake of fruit among children has improved slightly, the majority are still far from age-adjusted recommended intakes [19]. The low intake of fruits and vegetables is a missed opportunity to add under-consumed nutrients to diets [19].

Traditional dried fruit (those with no sugar added, such as raisins, prunes, dates, and figs) have been a staple of Mediterranean diets, valued for their sweetness and long-term stability. Traditional dried fruit has a similar nutrient profile as the original food and, as a result, dried fruit is typically a good source of fiber and potassium (Table 1). The consumption of dried fruit today is even less than fresh or canned; about 6.9 % of the adult population consume dried fruit [20, 21]. In an analysis of NHANES data from 1999–2004, raisins were the most commonly consumed dried fruit. Because they are used throughout the food system in products such as breads, muffins, cookies and cereals, raisins are consumed 6 times more than other dried fruits. In this study, dried fruit consumers were defined as those consuming 1/8 cup-equivalent dried fruit per day or more. Compared with non-consumers, dried fruit consumers had significantly lower intakes of solid fats/alcohol and added sugars and higher intakes of vegetables, meat, and soy products. Average energy intake of dried fruit consumers was 1038 kJ higher per day than non-consumers, yet they had significantly lower weight, BMI, waist circumference, and subscapular skinfold measurements, even after adjusting for lifestyle measures such as physical activity, TV/computer use, and smoking [21]. Additionally, adult dried fruit consumers had significantly higher intakes of fiber, vitamins A, C, E, thiamin, riboflavin, niacin, and folate. Vitamin B12 was the only vitamin that was not higher among dried fruit consumers [21]. Consumers of dried fruit had significantly higher intakes of calcium, phosphorus, magnesium, iron, zinc, copper, and potassium and significantly lower intakes of sodium [21].

**Table 1:** Average nutrient composition of most popular traditional dried fruits (per 100 g)

Dried Fruit^a,b^	Energy *Kcal*	Fat *g*	Carbohydrate *g*	Sugars *g*	Protein *g*	Fiber *g*	Calcium *mg*	Iron *mg*	Magnesium *mg*	Sodium *mg*	Potassium *mg*	Copper *mg*	Carotenoids *mg*	Total Phenol^c^ *mg GAE/100 g*
Apples^d^	243	0.4	66.0	57.2	0.9	8.7	14	1.4	16	87	450	0.19	0	324^e^
Apricots^d^	241	0.5	62.6	53.4	3.4	7.3	55	2.7	32	10	1162	0.34	2163	248^e^
Currants (Zante)	283	0.3	74.3	67.3	4.1	6.8	86	3.3	41	8	892	0.47	44	nd
Dates (deglet noor)	283	0.4	75.0	63.4	2.5	5.9	39	1.0	43	2	656	0.21	6	661
Dates (medjool)	277	0.2	75.0	66.5	1.8	6.7	64	0.9	54	1	696	0.36	112	572
Figs	249	0.9	63.9	47.9	3.3	9.8	162	2.3	68	10	680	0.29	38	960
Peaches^d^	239	0.8	61.3	41.7	3.6	8.7	28	4.0	42	7	996	0.36	2077	283^e^
Pears^d^	262	0.63	69.7	62.2	1.9	7.5	1.0	2.1	33	6	533	0.37	25	679^e^
Plums/prunes	240	0.38	63.9	38.1	2.2	7.1	43	0.9	41	2	732	0.28	55	938
Raisins	299	0.5	79.2	59.2	3.1	3.7	50	1.9	32	11	749	0.32	0	1065

*GAE* gallic acid equivalents; *nd* not determined

^a^Traditional dried fruit are defined as those with no added sugars, typically sun-dried or dried with minimal processing

^b^Nutrient information from US Department of Agriculture Nutrient Database Standard Reference #27

^c^Total phenol data from Alasalvar, C. and Shahidi, F. [29]

^d^ Sulfured

^e^Values calculated from fruit dried to 40 % moisture

The researchers also examined the association between dried fruit intake and diet quality using the Healthy Eating Index -2005 (HEI 2005) [21]. The HEI-2005 is an objective measure of diet quality developed and validated by the USDA. Dried fruit consumers had significantly higher total HEI 2005 scores (about 8 % higher) compared to non-consumers. As a group, dried fruit consumers also had significantly higher HEI component scores on fruit, dark green/orange vegetables and legumes, whole grains, milk, saturated fats, and empty calorie scores, compared to non-consumers.

#### Nuts

Tree nuts have been consumed for thousands of years, providing a concentrated source of energy, nutrients, and bioactive compounds, including unsaturated fatty acids [22]. While nutrient content varies among species, tree nuts generally are good to excellent sources of protein, dietary fiber, vitamins E and K, folate, magnesium, copper, and potassium and are rich in phytosterols and phenolic compounds (Table 2). Consumption of nuts is even less common than for fruit, with a small minority, about 6.8 % of the population, including nuts in their diets on a regular basis [23]. Mixed nuts containing peanuts (which are technically legumes) are the most commonly consumed nuts, walnuts the least. A recent analysis of data from NHANES 2005–2010 examined the nutrient adequacy of more than 14,000 adult tree nut consumers vs non-consumers, excluding pregnant and lactating women [24]. Tree nut consumers were identified as those consuming ≥ ¼ ounce/day. In this study, tree nut consumers were more likely to be non-Hispanic, older, have higher incomes, were less likely to smoke and were more physically active than non-consumers. Consumers of nuts also had significantly higher energy intakes, about 200 kcal more, on days that nuts were consumed, than non-consumers. It is interesting, however, that despite the significant increase in energy intake, there was no increase in BMI or waist circumference [24]. While tree nuts are an energy-rich food, estimates are that 55–75 % of the energy consumed is offset by dietary compensation and another 10–15 % is not absorbed [25, 26].

**Table 2:** Average nutrient composition of nuts (per 100 g)^1^

Nut	E *Kcal*	Fat *g*	SFA *g*	MUFA *g*	PUFA *g*	Protein *g*	Fiber *g*	Phytosterols *mg*	Calcium *mg*	Magnesium *mg*	Sodium *mg*	Potassium *mg*	Tocopherols *mg*	Carotenoids *mg*	Total Phenols *mg*	Flavonoids *mg*	Procyanidins *mg GAE/100 g*
Almonds	575	50.6	3.9	32.2	12.2	21.3	8.8	120	248	275	1	728	25	2	287	15	184
Brazil nuts	656	66.4	15.1	24.5	20.6	14.3	8.5	nd	160	376	3	659	4	nd	244	nd	nd
Cashews	553	46.4	9.2	27.3	7.8	18.2	5.9	158	37	292	12	660	1	nd	137	2	9
Hazelnuts	628	60.8	4.5	45.7	7.9	15	10.4	96	114	163	0	680	33	106	687	12	500
Macadamias	718	75.8	12.1	58.9	1.5	7.9	6	116	85	130	5	368	4	nd	126	nd	nd
Peanuts	567	49.7	6.9	24.6	15.7	23.7	3.1	220	92	168	18	705	8	nd	406	0,7	16
Pecans	691	72	6.2	40.8	21.6	9.2	8.4	102	70	121	0	410	4	55	1284	34	494
Pine nuts	673	68.4	4.9	18.8	34.1	13.7	3.7	141	16	251	2	597	6	nd	32	0,5	nd
Pistachios	557	44.4	5.4	23.3	13.5	20.6	9	214	107	121	1	1025	7	332	867	14	237
Walnuts	654	65.2	6.1	8.9	47.2	15.2	6.4	72	98	158	2	441	6	nd	1576	3	67

^1^Nutrient information from the US Department of Agriculture Nutrient Database Standard Reference #27 GAE: gallic acid equivalents nd: not determined

The analysis of NHANES 2005–2010 data also found that fewer consumers of tree nuts and tree nut butters had usual intakes (UI) of vitamins A, E and C, folate, calcium, iron, magnesium, and zinc that were below the Estimated Average Requirement (EAR) [24]. Consumers of tree nuts and tree nut butters were also more likely to have UIs above the Adequate Intakes (AIs) for dietary fiber and potassium. Only about 4 % of the non-consumer population achieved dietary fiber Adequate Intakes (AI) of 14 g per 1,000 per day, whereas among tree nut and nut butter consumers, almost one-third of the population reached the AI. The higher intake of fiber among nut consumers was of the same magnitude as for dried fruit consumers [21, 24]. Tree nut consumers also had a significantly higher potassium intake; 11.9 % were above the AI, compared to 1.9 % among non-consumers, though both groups remained well below the AI of 4,700 mg/day. Sodium intake was high in both groups. Diet quality, as measured by the HEI-2005, was significantly higher in tree nut consumers compared to non-consumers (Table 3). In addition, total fruit, dark green and orange vegetables, sodium and solid fat, alcohol and added sugars (SoFAAS) kilocalories component scores were higher in tree nut consumers than non-consumers.

**Table 3:** Changes in Diet Quality (HEI-2005) and Subcomponent Scores Associated with tree nut/dried fruit consumption in adults (19+ years)

	Diet Quality (HEI-2005) and Subcomponent Scores Associated with Tree nut or Dried Fruit Consumption in Adults (19+)^a^				Calculated Difference from Non-Consumer in each food category^b^	
	Tree Nut Consumer	Tree Nut Non-Consumer	Dried Fruit Consumer	Dried Fruit Non-Consumer	Tree Nut	Dried fruit
HEI Component Score	LS Mean (SE)		LS Mean (SE)			
Total Score (100)	61.2 (0.70)*	52.4 (0.30)	59.3 (0.50)*	49.4 (0.20)	8.8	9.9
Total Fruit (5)	2.5 (0.10)*	2.1 (0.03)	3.3 (0.08)*	2.0 (0.05)	0.4	1.3
Whole Fruit (5)	2.4 (0.09)*	2.0 (0.03)	3.4 (0.07)*	1.8 (0.04)	0.4	1.7
Total Vegetable (5)	3.1 (0.09)	3.0 (0.03)	3.0 (0.06)	3.0 (0.03)		
Dark Green & Orange Vegetable (5)	1.7 (0.11)*	1.2 (0.03)	1.5 (0.09)*	1.2 (0.03)	0.4	0.3
Total Grain (5)	3.7 (0.06)*	4.2 (0.02)	4.3 (0.05)*	4.2 (0.02)	‐0.5	0.2
Whole Grain (5)	1.4 (0.11)*	1.1 (0.02)	1.8 (0.06)*	0.9 (0.02)	0.3	0.9
Milk (10)	5.1 (0.13)	5.2 (0.05)	5.4 (0.16)*	4.7 (0.06)		0.6
Meat & Beans (5)	9.2 (0.08)*	8.0 (0.04)	8.0 (0.13)	8.2 (0.04)	1.2	
Oils (10)	8.7 (0.08)*	6.3 (0.05)	5.7 (0.15)	5.3 (0.05)	2.4	
Saturated Fat	6.2 (0.18)	5.8 (0.06)	6.8 (0.14)*	5.9 (0.06)		1.0
Sodium (10)	4.4 (0.15)*	3.3 (0.04)	4.9 (0.14)*	4.2 (0.05)	1.1	0.7
SoFAAS Calories (20)	12.8 (0.32)*	10.2 (0.11)	11.1 (0.24)*	8.2 (0.14)	2.7	3.0

^a^Sample weighted regression analyses with the following covariates: gender, age, race/ethnicity, and energy. Dried fruit data from NHANES 1999-2004. Tree nut data from NHANES 2005-2010. Data adapted from 21, 24)

^b^Shading indicates potential complementary contribution of tree nuts and dried fruit LS Mean: Least square mean; SE: Standard error; SoFAAS: Solid fat, alcohol, and added sugars

*Difference between non-consumer and consumer in each food category (tree nut or dried fruit) *p* < 0.01

#### Potential dietary benefits of pairing nuts and dried fruit

Nuts and dried fruit have a complementary portfolio of nutrients. There is a cultural tradition of pairing them as snacks or in prepared foods. Yet, no study has evaluated the impact of combining nuts and dried fruit on the nutritional quality of the diet. Table 3 shows the potential effect of pairing tree nuts and dried fruit on diet quality, by comparing the differences in HEI scores between each consumption group and the corresponding non-consumption group. While nuts and dried fruit consumers have similar increases in HEI scores over non-consumers, there is no complete overlap of these groups (shaded areas). Combining these foods may thus further increase HEI scores. This is important because small changes in the diet can have a significant impact on health. A study calculated the potential public health impact on CVD mortality of replacing a unhealthy snacks (e.g., crisps, candy, cakes) with healthy ones (e.g., fruits, dried fruits, unsalted nuts). They found that simply replacing one unhealthy snack per day with a healthy one might prevent approximately 6000 cases of CVD per year in the UK [27]. The Baltimore Longitudinal Study on Aging reported that CHD was approximately 75 % lower among men with both low saturated fat intake and high fruit and vegetable intake. This combination was considerably more protective than each behavior alone [28].

### Dried fruit and cardiometabolic health

The limited number of clinical studies on dried fruit and cardiometabolic health suggests that it can lower the postprandial insulin response, modulate sugar absorption (Glycemic Index), promote satiety, and have a beneficial effect on blood pressure (BP) [29, 30]. An analysis of three large prospective longitudinal cohort studies, the Nurses’ Health Study, the Nurses’ Health Study II and the Health Professionals Follow-up Study, with a total of more than 3 million person years, found that associations of fruit consumption with the risk of developing type 2 diabetes differed significantly among individual fruits [31]. The results showed that each incremental intake of 3 servings a week of certain whole fruits, including grapes or raisins, dried plums, apples or pears, grapefruits, and blueberries, was associated with a significant reduction in risk of developing type 2 diabetes. Not all fruits were associated with a reduction in risk.

**Fig. 1:**
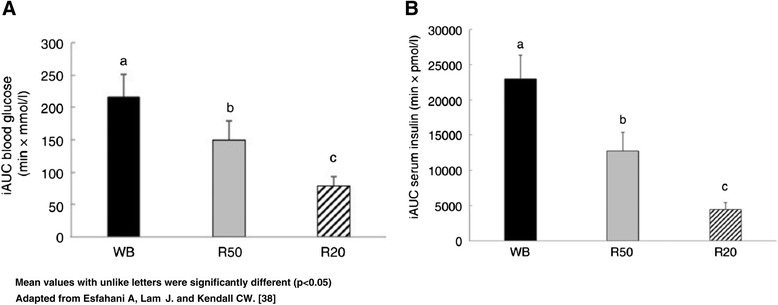
Incremental AUC (iAUC) for glucose (**A**) and insulin (**B**) after consumption of 3 meals containing 50, 50 and 20 g of available carbohydrate from white bread (WB), raisins (R50) and raisins (R20), respectively

Like many fresh fruits, dried fruits have a low to moderate glycemic index (GI) and a single serving exerts a low glycemic load (GL) [32]. The GI measures the blood-glucose-raising effect of an amount of a carbohydrate-containing food that provides 50 g of carbohydrates [33]. The GL predicts the blood sugar response to a standard serving of a food [34]. Diets with a low GL help manage blood sugar and lipid levels and may lower the risk of developing diabetes [35, 36]. The GI of raisins was first determined to be 64 ± 11 (glucose = 100) in a study with 6 healthy, non-diabetic individuals [33]. A later study measured raisin GI and Insulin Index in 10 sedentary adults, 10 pre-diabetic individuals, and 11 endurance athletes [37]. The GI was 49.4 ± 7.4, 49.6 ± 4.8, and 62.3 ± 4.8, respectively. The Insulin Index was correspondingly low as well. This is important because high fasting and/or postprandial insulin levels can increase cholesterol synthesis and impair fat mobilization from adipose tissue. In a more recent study, the GI, Insulin Index, and glycemic response of raisins was assessed in 10 healthy subjects [38]. The GI was 49 ± 4 with a corresponding low Insulin Index. The study also showed that a serving of raisins (28 g) exerts a low GL. Compared to an equal amount of carbohydrate from white bread, raisins had a significantly lower postprandial glucose and insulin response. This indicates that raisins can modulate the glycemic response (Fig. 1). A single-crossover study found little difference between the glycemic responses to 100-cal servings of grapes or raisins among subjects with type 2 diabetes. Insulin AUC value for raisins was lower than that for grapes (2970 ± 849 and 3960 ± 1370), but the difference was not significant [39].

Differences in blood glucose responses were found when subjects were given a nut and dried fruit snack (raisins and peanuts) or processed snacks (chocolate-coated candy bar or a cola drink with crisps) [40]. Peak glucose concentrations tended to be higher after the processed snacks than after the peanuts raisin snacks and plasma insulin levels were significantly lower, despite having similar amounts of total carbohydrate, sugar, fat, and protein. Factors thought to contribute to raisins’ lower glycemic response were the food matrix, the presence of tartaric acid, and the type of sugar present. Because of their fat content, adding nuts to a dried fruit snack would be expected to lower the glycemic response. Pistachios consumed with a carbohydrate containing food (pasta, parboiled rice, mashed potatoes) reduced in a dose-dependent manner the total postprandial response by 20 – 30 % [41].

Eating fruit has been associated with reductions in body weight. The association between raisin intake (together with other dried fruits) and body weight was examined in a cross-sectional study (*n* = 13292 adults). Individuals consuming more than 20 g dried fruit per day had higher energy intakes than those who consumed less, but had a lower mean body weight, body mass index, and waist circumference. Moreover, after adjusting for potential confounders, prevalence of overweight/obesity and abdominal obesity was lower for those in the group consuming more dried fruit [21].

Intervention studies with raisins show that consumption may have a beneficial effect on blood lipids. In a study on hyperlipidemic subjects, a diet providing 126 g of raisins per day reduced total cholesterol and LDL cholesterol by 13 and 16 % respectively, but did not affect plasma triglyceride levels [42]. After consuming Mediterranean-style diets that provided 84 g of raisins and mixed nuts and olive oil daily for 4 weeks, cholesterol and LDL cholesterol were 9 and 15 % lower, respectively, in hypercholesterolemic men [43, 44].

Anderson found that among mildly hyperglycemic and hypertensive subjects (*n* = 31), eating raisins three times per day over a 12-week period, significantly reduced post-meal glucose and HbA1C levels when compared with subjects (*n* = 15) eating popular commercial non-fruit snacks (e.g. crackers, cookies) of the same caloric content [45]. Hypertension is a primary risk factor for cardiovascular disease. Raisin consumption was also associated with statistically significant reductions in both systolic and diastolic BP. No significant changes in body weight were observed within or between groups.

A 12-week randomized study (*n* = 57) evaluated the effect of routine intake of either raisins or processed snacks on glucose levels and other cardiovascular risk factors among patients with type 2 diabetes. In this study, those who consumed raisins had a significant 23 % reduction in postprandial glucose levels (*p* = 0.024) compared to those consuming processed snacks. Also, compared to snack consumption, those who consumed raisins had a 19 % reduction in fasting glucose and a non-significant reduction of 0.12 % in HbA1c. Compared to processed snacks, those who consumed raisins had a significant reduction in systolic BP, but not in diastolic BP. Body weight, BMI, waist circumference, fasting insulin, homeostatic model assessment of insulin resistance, total cholesterol, LDL, HDL, triglyceride, or non-HDL cholesterol levels did not differ with treatment [46] (Table 4). These results highlight the impact that the composition of snacks can have on blood sugar.

**Table 4:** Cardiometabolic effects of raisins compared to processed snacks in patients with hyperglycemia and in patients with type 2 diabetes (T2D), summary of two similarly designed 12 week studies [45, 46]

Metabolic parameter	Patients with Hyperglycemia but not T2D	Patients with T2D
Postprandial glucose (%)	Raisins: 16 % ↓ (*p* = 0.033)	Raisins: 23 % ↓ (*p* = 0.024)
Postprandial glucose (mg/dL)	Raisins: ↓ by 13.5 mg/dL (*p* = 0.09)	Raisins: ↓ by 36 mg/dL (*p* = 0.07)
Fasting glucose (%)	Raisins: ↓ by 8 % (*p* = 0.75)	Raisins: ↓ by 19 % (*p* = 0.06)
HbA1c (%)	Raisins: ↓ by 0.08 % (*p* = 0.17)	Raisins: ↓ by 0.12 % (*p* = 0.62)
Systolic Blood Pressure (%)	Raisins: ↓ by 4.8 % (*p* = 0.047)	Raisins: ↓ by 7.5 % (*p* = .031)
Systolic Blood Pressure (mmHg)	Raisins: ↓ by 6.5 mmHg (*p* = 0.047)	Raisins: ↓ by 8.7 mmHg (*p* = 0.035)
Other metabolic parameters	Raisins did not improve diastolic BP, weight, body mass index, waist circumference, fasting insulin, total cholesterol, LDL, HDL and non HDL-cholesterol, triglyceride	Raisins did not improve diastolic BP, weight, body mass index, waist circumference, fasting insulin, total cholesterol, LDL, HDL and non HDL-cholesterol, triglyceride

In another study, a group of patients with well-controlled type 2 diabetes were instructed to eat fewer fruits and vegetables than the recommended five servings per day and were given two servings of Corinthian raisins or snacks of similar energy density for 24 weeks. Consuming raisins significantly lowered diastolic BP and increased total antioxidant potential compared to levels at baseline. Raisins did not affect body weight, glycemic control, lipid profile, or C-reactive protein [47].

Fructose comprises approximately 50 % of the available carbohydrate content of raisins [38]. When recommending increased consumption of fruit, especially dried fruit, concerns have been raised about the effects of fructose on cardiometabolic health. Several systematic reviews and meta-analyses have found that isocaloric exchange of fructose for other carbohydrates has no effect on several cardiometabolic risk factors. One meta-analysis found that isocaloric substitution of fructose had no effect on long-term glycemic control in individuals with diabetes [48]. Isocaloric substitution of fructose for other carbohydrates does not increase postprandial triglycerides or other established lipid targets for CVD [49]. The association of fructose consumption with hypertension and weight gain has also been examined and no relationship found [50]. Fructose that provides excess energy has been associated with increased body weight. However, this may be due to the excess calories, rather than excess fructose, specifically [50].

Proposed World Health Organization (WHO) Guidelines on sugar consumption recommend limiting added sugar intake to less than 10 % of total energy intake per day and that reducing intake further, to less than 5 % of energy intake, would have additional health benefits [51]. Similarly, the 2015 DGAs has recommended a goal for the general population of a maximum of 10 % of total calories from added sugar per day [16]. The limits apply to all sugars (glucose, fructose, sucrose) added to foods by manufacturers and consumers. It also includes sugars present in honey, syrups, fruit juices, and fruit concentrates, but not the sugars naturally present in whole fruit, including traditional dried fruit.

### Nuts and cardiometabolic health

Nut consumption has been associated with a reduced risk for cardiovascular disease and diabetes [12, 14, 52, 53]. While the evidence is stronger for cardiovascular disease protection, these conditions are related and are both believed to be inflammatory in nature. Prospective studies [54, 55] and clinical trials have found nut consumption to be associated with decreases in inflammatory markers [5, 13, 56].

Large prospective studies have found an association between nut consumption with a lower risk of fatal coronary heart disease (CHD) [52]. The more frequent the intake, up to 5 or more times per week, the lower the risk. Lower total mortality has also been associated with frequency of nut consumption [12, 14, 57]. Despite a documented increase in caloric intake among nut consumers, a meta-analysis of randomized clinical trials found no association between nut consumption and increased body weight, BMI or weight circumference [58]. The PREDIMED study, which evaluated the effects of a Mediterranean Diet, rich in nuts (30 g/day) or extra virgin oil (50 g/day), versus a lower fat diet, reported reduced cardiovascular events (by 30 % after 4.7 years) in individuals at very high risk of CVD who consumed the Mediterranean diets [59]. In this study, the risk of stroke was also reduced in the two Mediterranean–diet groups [60]. A systematic review of twenty prospective cohort studies involving 467,389 participants, comparing highest to lowest nut consumers found that nut consumption was associated with a 44 % lower risk of total CVD, a 27 % risk of death from any type of CVD, a 34 % lower risk of all CHD, a 30 % lower risk of CHD mortality and a 47 % lower risk of sudden cardiac death [12].

However, not all studies have been consistent in finding an association between nut consumption and a reduction in disease risk. Two recent meta-analyses of prospective studies found that while a higher consumption of nuts (one serving per day or more) was associated with reduced risk of coronary artery disease (CAD) and hypertension, no association was found between nut consumption and stroke or type 2 diabetes [61, 62]. A German cohort study of 26 285 participants also failed to find an association between nut consumption and the risk of stroke [63]. However, a more recent meta-analysis of 9 prospective cohorts with 476,181 participants found a significant inverse association between nut consumption and stroke mortality among women [64]. Similarly, a meta-analysis of 8 prospective cohort studies found that a high intake of nuts, but not legumes, was inversely associated with stroke risk [65]. Another meta-analysis of prospective studies found that consumption of nuts (>2 servings a week) was inversely associated with hypertension risk, but not with risk of type 2 diabetes [66]. The Multi-Ethnic Study of Atherosclerosis (MESA) and the Nurses’ Health Study evaluated the effect of nut consumption on several inflammatory markers, with mixed results. The MESA trial found an association between nut consumption and lower CRP, IL-6, and fibrinogen levels [67]. The Prevención con Dieta Mediterránea (PREDIMED) study found lower ICAM1, but no differences in the other inflammatory markers were found [22]. The Moli-sani prospective study of a Mediterranean population found a reduction of inflammatory markers (CRP, platelet count, and neutrophil to lymphocyte ratio) among nut consumers, but the impact on CVD was limited to a non-significant trend [13]. The Nurses’ Health Study found no association between nut consumption and any of the inflammatory markers measured [68].

The majority of short-term clinical trials, conducted in otherwise healthy adults with type 2 diabetes or metabolic syndrome, are in agreement that an improvement in glucose and insulin metabolism occurs in the postprandial state with the consumption of 28–90 g of almond, pistachios or mixed nuts [41, 69–72].

The results from several long-term clinical trials conducted with different populations—some were obese, some had type 2 diabetes—and fed different types of nuts (almonds, walnuts, pistachios, almonds, and mixed nuts) are less consistent. Some found no effect of nut consumption on glucose and insulin metabolism [73–77]. Others found significant reductions in glucose, insulin, and HBA1c [78, 79] when two ounces of nuts replaced carbohydrate foods in the diet, suggesting health benefits [80]. A systematic review and meta-analysis of 47 RCT with subjects diagnosed with dyslipidemia, metabolic syndrome, or type 2 diabetes mellitus, showed significant reductions in triglycerides and fasting blood glucose among those given the diets supplemented with tree nuts (~50 g/day), compared with control diet interventions [81]. As with the majority of studies, nut consumption had no effect on waist circumference, or high-density lipoprotein cholesterol. BP was unaffected. A more recent systematic review and meta-analysis of 21 randomized controlled clinical trials to estimate the effect of nuts on BP found that total nut consumption lowered systolic BP in participants without type 2 diabetes. Pistachios appeared to have the strongest effect in reducing both systolic and diastolic BP [10].

The beneficial effects of pistachios on cardiometabolic risk factors have recently become the focus of research efforts. Pistachios are higher in β-carotene, lutein and γ-tocopherol, than other tree nuts, making them good sources of antioxidants, and they are higher in phytosterols, which are proven to reduce plasma total cholesterol and LDL-C levels. A clinical feeding trial was conducted in 54 middle-aged, overweight or obese subjects (average for the two groups, BMI 28.76–28.90) with pre-diabetes, given 57 g of pistachios daily or a control diet for 4 months, each with a 2-week washout period in between [5]. Significant decreases in glucose, plasma insulin, HOMA-IR, HbA1c, fibrinogen, and platelet factor 4 and a significant increase in glucagon-like peptide-1 were observed as a result of the pistachio diet (Table 5). Waist circumference, BMI, and weight did not change. While no changes in the classic markers of cardiovascular disease, such as total cholesterol, LDL-C, and HDL-C, were observed, oxidized LDL-C was significantly lower when the diet was supplemented with pistachios.

**Table 5:** Beneficial effect of pistachio consumption on glucose metabolism, insulin resistance, inflammation and related metabolic risk markers: results from a randomized clinical trial

	Pistachio diet		Control diet		Treatment effect
Characteristics	Baseline	Change	Baseline	Change	*P*-value
Fasting plasma glucose (mg/dL)	116.24 (112.37, 120.11)	-­5.17 (-­8.14, -­2.19)*	108.06 (104.27, 111.84)	6.72 (4.38, 9.07)	<0.001
Fasting plasma insulin (mU/mL)	14.36 (12.65, 16.07)	-­2.04 (-­3.17, -­0.92)*	11.44 (9.81, 13.07)	2.51 (1.02, 4.00)	<0.001
HOMA-­IR	4.22 (3.66, 4.77)	-­0.69 (-­1.07, -­0.31)*	3.10 (2.64, 3.56)	0.97 (0.49, 1.44)	<0.001
HOMA-­BCF	98.22 (86.35, 110.09)	-­3.46 (-­11.45, 4.53)	96.76 (78.45, 115.07)	-­0.25 (-­9.65, 9.16)	0.620
HbA1c (%)	5.92 (5.82, 6.02)	­0.03 (-­0.12, 0.05)	5.87 (5.75, 5.99)	0.03 (-­0.03, 0.10)	0.130
Fibrinogen (ng/mL)	71.18 (65.62, 76.75)	-­2.24 (-­5.94, 1.46)	65.13 (60.45, 69.81)	3.24 (-­0.19, 6.67)	0.019
Tissue Factor (pg/mL)	195.71 (143.16, 248.26)	16.33 (-­10.60, 43.27)	225.57 (169.88, 281.26)	-­14.46 (-­40.35, 11.43)	0.162
PAI-­1 (pg/mL)	158.37 (134.65, 182.10)	13.26 (-­13.81, 40.33)	177.42 (136.40, 218.43)	-­12.91 (-­42.41, 16.59)	0.146
Von Willebrand factor (ng/mL)	0.61 (0.47, 0.75)	0.27 (0.00, 0.55)	0.99 (0.59, 1.39)	-­0.04 (-­0.53, 0.45)	0.149
Platelet Factor 4 (ng/mL)	0.20 (0.07, 0.32)	-­0.07 (-­0.13, -­0.02)	0.12 (0.09, 0.15)	0.00 (-­0.02, 0.02)	0.014
Thromboxane B2 (ng/mL)	2.20 (1.60, 2.80)	-­0.18 (-­0.55, 0.19)	2.20 (1.69, 2.71)	0.13 (-­0.33, 0.58)	0.306
Gastric Inhibitory Polypeptide (pg/mL)	32.55 (26.99, 38.11)	-­0.04 (-­4.17, 4.09)	34.19 (29.17, 39.21)	-­1.31 (-­5.08, 2.46)	0.613
Glucagon-­Like Peptide-­1 (pg/mL)	46.62 (37.24, 56.00)	4.09 (1.25, 6.94)*	47.40 (37.77, 57.04)	-­0.59 (-­2.98, 1.80)	0.009
C-­peptide (ng/mL)	1.83 (1.68, 1.98)	-­0.06 (-­0.18, 0.06)	1.75 (1.60, 1.91)	0.01 (-­0.11, 0.14)	0.338
Resistin (pg/mL)	105.70 (89.89, 121.50)	2.29 (-­7.14, 11.73)	108.63 (89.55, 127.71)	4.19 (-­21.01, 29.39)	0.669

Adapted from Hernandez-­Alonso, P. et al [5]

All values are means (95 % CI). Intra-­group analysis was assessed by paired t-­test. Basal-­adjusted changes between groups were analysed using adjusted ANOVA of repeated measurements

* Significantly difference (*P* < 0.05) between baseline and final in a certain intervention period

There is limited evidence about the effect of nuts on modulating lipoprotein size and composition. A randomized, cross-over, controlled feeding study (a balanced order sequence of 4 weeks followed by a 2-week compliance break) was conducted to evaluate the cholesterol-lowering effects of diets containing either 1 or 2 servings of pistachios as part of an isoenergetic diet (10 and 20 % of energy, respectively) [82]. Consuming 2 servings of pistachios per day significantly decreased small and dense LDL and lowered the TAG:HDL ratio. In addition, both pistachio diets raised levels of α-1 and α-2 HDL particles. More recently, a randomized cross-over, controlled, feeding study was conducted to investigate the effect of pistachio consumption (57 g/day) on lipoprotein subclasses in pre-diabetic individuals. There was a shift in lipoprotein size and particle profile to a less atherogenic pattern. This suggests that pistachios may play a beneficial role in CVD, other than an effect on the classic lipid factors of cardiovascular risk [83].

Taken together, these results suggest that regular consumption of pistachios, as part of a moderate-fat diet, could have important glucose- and insulin-lowering effects, promote a healthier metabolic profile, and reverse certain metabolic consequences of pre-diabetes.

Obesity is an importan risk factor for CVD and type 2 diabetes. There is a perception among consumers that including nuts in the diets on a regular basis will result in weight gain. However, the evidence base does not support this perspective. The results of a meta-analysis of 33 randomized clinical trials found no association between consumption of nuts and body weight (0.47 kg), BMI (0.40), or weight circumference [58]. In fact, there was a non-significant decrease in all three—body weight (0.47 kg), BMI (0.40), and weight circumference (1.25 cm).

### Possible mechanisms

Nuts provide fiber, MUFA and PUFA and are low in SFA. Many are rich sources of tocopherols, phytosterols, and omega-3 fatty acids (alpha linolenic acid). These nutrients may improve inflammatory status, decrease total and LDL-cholesterol, reduce lipid peroxidation, and modulate endothelial function, resulting in beneficial effects on cardiovascular risk factors [52, 53, 84]. Nuts, particularly pistachios, have a high L-arginine content a precursor of endogenous vasodilator nitric oxide, which may contribute to vascular reactivity [53]. Nuts are also high in polyphenol antioxidants, which may act by binding to lipoproteins, thus inhibiting the oxidative processes that lead to atherosclerosis. Finally, nuts are a good source of many micronutrients that play a role in in CVD risk, including low sodium, high magnesium, potassium and calcium [53]. Individually or in combination, these compounds also have the potential to affect glucose metabolism. In diabetes, lymphocytes are hyperreactive, taking in large amounts of glucose. This increased glucose uptake by lymphocytes may lead to immune hyperactivity and inflammation [85]. A significant decrease in cellular glucose transport (CGT) activity has been demonstrated with nut consumption, as well [5].

Several biological mechanisms could explain why, despite a relatively high calorie and fat content, consumption of nuts is not associated with weight gain. Nuts are rich in unsaturated fatty acids, which may have a greater thermogenic effect than saturated fats, resulting in less fat storage [5]. Nuts are more satiating than many other foods, due to their energy density, fiber, and protein contents and consumption results in fewer calories consumed at subsequent eating occasions [86]. In addition, not all of the fat in nuts is absorbed following consumption, resulting in an overestimation of their caloric contribution to the diet [87]. This has been demonstrated in almonds [88] and pistachios [89].

Although several studies have found no significant changes in classic lipid profiles as a result of adding nuts to the diet, the antiatherogenic properties of nuts could be the result of alterations in various lipoprotein subclasses, including an increase in the size of LDL particles. A crossover study of 18 hyperlipidemic subjects fed walnuts (48 g) in addition to their habitual diets for 6 weeks resulted in no change in total cholesterol and apolipoprotein B concentrations [6]. Although there was no change in these lipid concentrations, the distribution of lipoprotein subfractions was altered. The addition of walnuts decreased the amount of cholesterol in the small LDL fractions and concentrations of apolipoprotein A-1 increased, indicative of a decreased risk for CHD.

Dried fruits are very low in sodium and are a particularly significant source of potassium and fiber. On a per serving basis, traditional dried fruit can deliver over 9 % of the Daily Value of these nutrients, depending on the fruit. Both of these nutrients play an important role in reducing the risk of cardiovascular disease, hypertension, and stroke [90–92]. Dried fruits are also excellent sources of polyphenols and phenolic acids [93]. These make up the largest group of plant bioactive compounds in the diet and appear to be responsible, at least in part, for the health benefits associated with the consumption of diets abundant in fruits and vegetables [94–96].

Increasing nuts and traditional dried fruit intake may displace intake of less healthy foods/snacks that are high in sodium or high in refined sugar, reducing glycemic load and possibly affecting cardiometablic risk factors. The spectrum of dietary antioxidants from nuts and dried fruits (tocopherols, carotenoids, polyphenols and phenolic acids) may lower overall oxidative stress by scavenging or neutralizing oxidant species and enhancing endogenous antioxidant defenses against metabolic impairment [3, 60]. Nuts and dried fruit are nutrient-rich foods, which provide micronutrients and bioactives that are individually associated with lower risk of CVD risk and metabolic disorders. The pairing of these foods thus would benefit many risk factors and physiological pathways.

## Conclusions

Only a small percentage of the US population regularly consumes tree nuts or dried fruit, yet tree nuts, along with dried fruit, are among the foods associated with some of the largest shifts toward a higher HEI score. As shown in the NHANES data, consumers of tree nuts and dried fruit had HEI scores of about 60, an improvement over the average HEI of the U.S. diet, which is about 50, but there is still enormous room for improvement. Increasing the intake of dried fruits and tree nuts, would improve nutrient intakes and the quality of the American diet.

Consumption of both nuts and dried fruit has been associated with decreases in waist circumference and BMI. A limited number of clinical studies on raisins and cardiometabolic health suggest that they can lower the postprandial insulin response, modulate sugar absorption, and have a beneficial effect on BP. Additional longitudinal studies are needed to confirm associations of dried fruit intake on these parameters. A larger body of evidence has suggested that regular consumption of nuts, such as pistachios, as part of a moderate-fat diet, could have important glucose- and insulin-lowering effects, promote a healthier metabolic profile, and reverse certain metabolic consequences of pre-diabetes.

Nuts and dried fruit have a complementary set of nutrients. Both are shelf-stable, portable, and accessible and, in many instances, they are consumed together as snacks or prepared foods. Yet, no study has evaluated the nutritional or health impact of nuts and dried fruits consumed together. Emerging data support the potential of pairing tree nuts and dried fruit, such as pistachios and raisins, as a way to reduce cardiometabolic risk factors, improve glycemic control and decrease the risk of developing diabetes and/or cardiovascular disease.

## References

[1] Sleiman D, Al-Badri MR, Azar ST (2015). Effect of mediterranean diet in diabetes control and cardiovascular risk modification: a systematic review. Front Public Health.

[2] USDA (2015). Scientific Report of the 2015 Dietary Guidelines Advisory Committee.

[3] Grosso G, Marventano S, Yang J, Micek A, Pajak A, Scalfi L, Galvano F, Kales SN. A Comprehensive Meta-analysis on Evidence of Mediterranean Diet and Cardiovascular Disease: Are Individual Components Equal? Crit Rev Food Sci Nutr. 2015 Nov 3:0. [Epub ahead of print] 10.1080/10408398.2015.1107021 26528631

[4] Li M, Fan Y, Zhang X, Hou W, Tang Z (2014). Fruit and vegetable intake and risk of type 2 diabetes mellitus: meta-analysis of prospective cohort studies. BMJ Open.

[5] Hernandez-Alonso P, Salas-Salvado J, Baldrich-Mora M, Juanola-Falgarona M, Bullo M (2014). Beneficial effect of pistachio consumption on glucose metabolism, insulin resistance, inflammation, and related metabolic risk markers: a randomized clinical trial. Diabetes Care.

[6] Almario RU, Vonghavaravat V, Wong R, Kasim-Karakas SE (2001). Effects of walnut consumption on plasma fatty acids and lipoproteins in combined hyperlipidemia. Am J Clin Nutr.

[7] Hudthagosol C, Haddad EH, McCarthy K, Wang P, Oda K, Sabate J (2011). Pecans acutely increase plasma postprandial antioxidant capacity and catechins and decrease LDL oxidation in humans. J Nutr.

[8] West SG, Gebauer SK, Kay CD, Bagshaw DM, Savastano DM, Diefenbach C (2012). Diets containing pistachios reduce systolic blood pressure and peripheral vascular responses to stress in adults with dyslipidemia. Hypertension.

[9] Gebauer SK, West SG, Kay CD, Alaupovic P, Bagshaw D, Kris-Etherton PM (2008). Effects of pistachios on cardiovascular disease risk factors and potential mechanisms of action: a dose-response study. Am J Clin Nutr.

[10] Mohammadifard N, Salehi-Abargouei A, Salas-Salvado J, Guasch-Ferre M, Humphries K, Sarrafzadegan N (2015). The effect of tree nut, peanut, and soy nut consumption on blood pressure: a systematic review and meta-analysis of randomized controlled clinical trials. Am J Clin Nutr.

[11] Nishi SK, Kendall CW, Bazinet RP, Bashyam B, Ireland CA, Augustin LS (2014). Nut consumption, serum fatty acid profile and estimated coronary heart disease risk in type 2 diabetes. Nutr Metab Cardiovasc Dis.

[12] Mayhew AJ, de Souza RJ, Meyre D, Anand SS, Mente A (2016). A systematic review and meta-analysis of nut consumption and incident risk of CVD and all-cause mortality. Br J Nutr.

[13] Bonaccio M, Di Castelnuovo A, De Curtis A, Costanzo S, Bracone F, Persichillo M (2015). Nut consumption is inversely associated with both cancer and total mortality in a Mediterranean population: prospective results from the Moli-sani study. Br J Nutr.

[14] Grosso G, Yang J, Marventano S, Micek A, Galvano F, Kales SN (2015). Nut consumption on all-cause, cardiovascular, and cancer mortality risk: a systematic review and meta-analysis of epidemiologic studies. Am J Clin Nutr.

[15] Kasliwal RR, Bansal M, Mehrotra R, Yeptho KP, Trehan N (2015). Effect of pistachio nut consumption on endothelial function and arterial stiffness. Nutrition.

[16] US Department of Health and Human Services, US Department of Agriculture (2015). 2015–2020 Dietary Guidelines for Americans.

[17] Centers for Disease Control and Prevention (2010). Morbidity and Mortality Weekly Report, State-specific trends in fruit and vegetable consuptin among adults—United States, 2000–2009.

[18] Grimm KABH, Scanlon KS (2010). State-specific trends in fruit and vegetable consumption among adults—United States, 2000–2009. Morb Mortal Wkly Rep.

[19] Kim SA, Moore LV, Galuska D, Wright AP, Harris D, Grummer-Strawn LM (2014). Vital signs: fruit and vegetable intake among children - United States, 2003-2010. MMWR Morb Mortal Wkly Rep.

[20] Bachman JL, Reedy J, Subar AF, Krebs-Smith SM (2008). Sources of food group intakes among the US population, 2001-2002. J Am Diet Assoc.

[21] Keast DR, O’Neil CE, Jones JM (2011). Dried fruit consumption is associated with improved diet quality and reduced obesity in US adults: National Health and Nutrition Examination Survey, 1999-2004. Nutr Res.

[22] Salas-Salvado J, Casas-Agustench P, Salas-Huetos A (2011). Cultural and historical aspects of Mediterranean nuts with emphasis on their attributed healthy and nutritional properties. Nutr Metab Cardiovasc Dis.

[23] O’Neil CE, Fulgoni VL, Nicklas TA (2015). Tree Nut consumption is associated with better adiposity measures and cardiovascular and metabolic syndrome health risk factors in U.S. Adults: NHANES 2005-2010. Nutr J.

[24] O’Neil CE, Nicklas TA, Fulgoni VL (2015). Tree nut consumption is associated with better nutrient adequacy and diet quality in adults: National Health and Nutrition Examination Survey 2005-2010. Nutrients.

[25] Mattes RD, Dreher ML (2010). Nuts and healthy body weight maintenance mechanisms. Asia Pac J Clin Nutr.

[26] Mattes RD (2008). The energetics of nut consumption. Asia Pac J Clin Nutr.

[27] Lloyd-Williams F, Mwatsama M, Ireland R, Capewell S (2009). Small changes in snacking behaviour: the potential impact on CVD mortality. Public Health Nutr.

[28] Tucker KL, Hallfrisch J, Qiao N, Muller D, Andres R, Fleg JL (2005). The combination of high fruit and vegetable and low saturated fat intakes is more protective against mortality in aging men than is either alone: the Baltimore Longitudinal Study of Aging. J Nutr.

[29] Shahadi FTZ, Alasalvar CSF (2013). Raisins: Processing, Phytochemicals and Health Benefits. Dried Fruits, Phytochemicals and Health Effects.

[30] Furchner-Evanson A, Petrisko Y, Howarth L, Nemoseck T, Kern M (2010). Type of snack influences satiety responses in adult women. Appetite.

[31] Muraki I, Imamura F, Manson JE, Hu FB, Willett WC, van Dam RM (2013). Fruit consumption and risk of type 2 diabetes: results from three prospective longitudinal cohort studies. BMJ.

[32] ACaS F, ACaS F (2013). Composition, phytochemicals and beneficial health elffects of dried fruit: an overview. Dried Fruits, Phytochemicals and Health Effects.

[33] Jenkins DJ, Wolever TM, Taylor RH, Barker H, Fielden H, Baldwin JM (1981). Glycemic index of foods: a physiological basis for carbohydrate exchange. Am J Clin Nutr.

[34] Monro JA, Shaw M (2008). Glycemic impact, glycemic glucose equivalents, glycemic index, and glycemic load: definitions, distinctions, and implications. Am J Clin Nutr.

[35] Liu S, Willett WC, Stampfer MJ, Hu FB, Franz M, Sampson L (2000). A prospective study of dietary glycemic load, carbohydrate intake, and risk of coronary heart disease in US women. Am J Clin Nutr.

[36] Mirrahimi A, Chiavaroli L, Srichaikul K, Augustin LS, Sievenpiper JL, Kendall CW (2014). The role of glycemic index and glycemic load in cardiovascular disease and its risk factors: a review of the recent literature. Curr Atheroscler Rep.

[37] Kim Y, Hertzler SR, Byrne HK, Mattern CO (2008). Raisins are a low to moderate glycemic index food with a correspondingly low insulin index. Nutr Res.

[38] Esfahani A, Lam J, Kendall CW (2014). Acute effects of raisin consumption on glucose and insulin reponses in healthy individuals. J Nutr Sci.

[39] Wilson TAJ, Anderson KF, Heimerman RA, Larson MM, Freeman MR, Baker SE (2012). Glycemic response of type 2 diabetics to raisins. Food Nutr Sci.

[40] Oettle GJ, Emmett PM, Heaton KW (1987). Glucose and insulin responses to manufactured and whole-food snacks. Am J Clin Nutr.

[41] Kendall CW, Josse AR, Esfahani A, Jenkins DJ (2011). The impact of pistachio intake alone or in combination with high-carbohydrate foods on post-prandial glycemia. Eur J Clin Nutr.

[42] Spiller GABB (1998). Regular consumption of high fiber carbohydrate foods such as sun dried raisins and whole grain breads does not raise serum triglycerides. FASEB J.

[43] Spiller GABB, Farquhar JW (1996). Llipid-lowering effect of a Mediterranean-type diet, high in total and soluble fiber and monunsaturated fat. Circulation.

[44] Bruce BSG, Farquhar JW (1997). Effects of a plant-based diet rich in whole grains, sun-dried raisins and nuts on serum lipoproteins. Veg Nutr Int J.

[45] Anderson JW, Weiter KM, Christian AL, Ritchey MB, Bays HE (2014). Raisins compared with other snack effects on glycemia and blood pressure: a randomized, controlled trial. Postgrad Med.

[46] Bays H, Weiter K, Anderson J (2015). A randomized study of raisins versus alternative snacks on glycemic control and other cardiovascular risk factors in patients with type 2 diabetes mellitus. Phys Sportsmed.

[47] Kanellos PT, Kaliora AC, Tentolouris NK, Argiana V, Perrea D, Kalogeropoulos N (2014). A pilot, randomized controlled trial to examine the health outcomes of raisin consumption in patients with diabetes. Nutrition.

[48] Cozma AI, Sievenpiper JL, de Souza RJ, Chiavaroli L, Ha V, Wang DD (2012). Effect of fructose on glycemic control in diabetes: a systematic review and meta-analysis of controlled feeding trials. Diabetes Care.

[49] Chiavaroli L, de Souza RJ, Ha V, Cozma AI, Mirrahimi A, Wang DD (2015). Effect of Fructose on Established Lipid Targets: A Systematic Review and Meta-Analysis of Controlled Feeding Trials. J Am Heart Assoc.

[50] Sievenpiper JL, de Souza RJ, Mirrahimi A, Yu ME, Carleton AJ, Beyene J (2012). Effect of fructose on body weight in controlled feeding trials: a systematic review and meta-analysis. Ann Intern Med.

[51] Organization WH (2015). Guideline: Sugars intake for adults and children.

[52] Ros E (2010). Health benefits of nut consumption. Nutrients.

[53] Bullo M, Juanola-Falgarona M, Hernandez-Alonso P, Salas-Salvado J (2015). Nutrition attributes and health effects of pistachio nuts. Br J Nutr.

[54] Nettleton JA, Steffen LM, Mayer-Davis EJ, Jenny NS, Jiang R, Herrington DM (2006). Dietary patterns are associated with biochemical markers of inflammation and endothelial activation in the Multi-Ethnic Study of Atherosclerosis (MESA). Am J Clin Nutr.

[55] Salas-Salvado J, Garcia-Arellano A, Estruch R, Marquez-Sandoval F, Corella D, Fiol M (2008). Components of the Mediterranean-type food pattern and serum inflammatory markers among patients at high risk for cardiovascular disease. Eur J Clin Nutr.

[56] Casas-Agustench P, Lopez-Uriarte P, Bullo M, Ros E, Cabre-Vila JJ, Salas-Salvado J (2011). Effects of one serving of mixed nuts on serum lipids, insulin resistance and inflammatory markers in patients with the metabolic syndrome. Nutr Metab Cardiovasc Dis.

[57] Guasch-Ferre M, Bullo M, Martinez-Gonzalez MA, Ros E, Corella D, Estruch R (2013). Frequency of nut consumption and mortality risk in the PREDIMED nutrition intervention trial. BMC Med.

[58] Flores-Mateo G, Rojas-Rueda D, Basora J, Ros E, Salas-Salvado J (2013). Nut intake and adiposity: meta-analysis of clinical trials. Am J Clin Nutr.

[59] Eguaras S, Toledo E, Buil-Cosiales P, Salas-Salvado J, Corella D, Gutierrez-Bedmar M, Santos-Lozano JM, Aros F, Fiol M, Fito M, et al. Does the Mediterranean diet counteract the adverse effects of abdominal adiposity? Nutr Metab Cardiovasc Dis. 2015;25(6):569–74. 10.1016/j.numecd.2015.03.001 25921850

[60] Estruch R, Ros E, Martinez-Gonzalez MA (2013). Mediterranean diet for primary prevention of cardiovascular disease. N Engl J Med.

[61] Zhou D, Yu H, He F, Reilly KH, Zhang J, Li S (2014). Nut consumption in relation to cardiovascular disease risk and type 2 diabetes: a systematic review and meta-analysis of prospective studies. Am J Clin Nutr.

[62] Luo C, Zhang Y, Ding Y, Shan Z, Chen S, Yu M (2014). Nut consumption and risk of type 2 diabetes, cardiovascular disease, and all-cause mortality: a systematic review and meta-analysis. Am J Clin Nutr.

[63] di Giuseppe R, Fjeld MK, Dierkes J, Theoflylaktopoulou D, Arregui M, Boeing H (2015). The association between nut consumption and the risk of total and ischemic stroke in a German cohort study. Eur J Clin Nutr.

[64] Zhang Z, Xu G, Wei Y, Zhu W, Liu X (2015). Nut consumption and risk of stroke. Eur J Epidemiol.

[65] Shi ZQ, Tang JJ, Wu H, Xie CY, He ZZ (2014). Consumption of nuts and legumes and risk of stroke: a meta-analysis of prospective cohort studies. Nutr Metab Cardiovasc Dis.

[66] Guo K, Zhou Z, Jiang Y, Li W, Li Y (2015). Meta-analysis of prospective studies on the effects of nut consumption on hypertension and type 2 diabetes mellitus. J Diabetes.

[67] Jiang R, Jacobs DR, Mayer-Davis E, Szklo M, Herrington D, Jenny NS (2006). Nut and seed consumption and inflammatory markers in the multi-ethnic study of atherosclerosis. Am J Epidemiol.

[68] Li TY, Brennan AM, Wedick NM, Mantzoros C, Rifai N, Hu FB (2009). Regular consumption of nuts is associated with a lower risk of cardiovascular disease in women with type 2 diabetes. J Nutr.

[69] Jenkins DJ, Kendall CW, Josse AR, Salvatore S, Brighenti F, Augustin LS (2006). Almonds decrease postprandial glycemia, insulinemia, and oxidative damage in healthy individuals. J Nutr.

[70] Josse AR, Kendall CW, Augustin LS, Ellis PR, Jenkins DJ (2007). Almonds and postprandial glycemia--a dose-response study. Metabolism.

[71] Kendall CWEA, Josse AR, Augustin LS, Vidgen E, Jenkins DJ (2011). The glycemic effect of nut-enriched meals in healthy and diabetic subjects. Nutri Metab Cardiovasc Dis.

[72] Kendall CW, West SG, Augustin LS, Esfahani A, Vidgen E, Bashyam B (2014). Acute effects of pistachio consumption on glucose and insulin, satiety hormones and endothelial function in the metabolic syndrome. Eur J Clin Nutr.

[73] Lovejoy JC, Most MM, Lefevre M, Greenway FL, Rood JC (2002). Effect of diets enriched in almonds on insulin action and serum lipids in adults with normal glucose tolerance or type 2 diabetes. Am J Clin Nutr.

[74] Wien MA, Sabate JM, Ikle DN, Cole SE, Kandeel FR (2003). Almonds vs complex carbohydrates in a weight reduction program. Int J Obes Relat Metab Disord.

[75] Tapsell LC, Gillen LJ, Patch CS, Batterham M, Owen A, Bare M (2004). Including walnuts in a low-fat/modified-fat diet improves HDL cholesterol-to-total cholesterol ratios in patients with type 2 diabetes. Diabetes Care.

[76] Ma Y, Njike VY, Millet J, Dutta S, Doughty K, Treu JA (2010). Effects of walnut consumption on endothelial function in type 2 diabetic subjects: a randomized controlled crossover trial. Diabetes Care.

[77] Li Z, Song R, Nguyen C, Zerlin A, Karp H, Naowamondhol K (2010). Pistachio nuts reduce triglycerides and body weight by comparison to refined carbohydrate snack in obese subjects on a 12-week weight loss program. J Am Coll Nutr.

[78] Tapsell LC, Batterham MJ, Teuss G, Tan SY, Dalton S, Quick CJ (2009). Long-term effects of increased dietary polyunsaturated fat from walnuts on metabolic parameters in type II diabetes. Eur J Clin Nutr.

[79] Wien M, Bleich D, Raghuwanshi M, Gould-Forgerite S, Gomes J, Monahan-Couch L (2010). Almond consumption and cardiovascular risk factors in adults with prediabetes. J Am Coll Nutr.

[80] Jenkins DJ, Kendall CW, Banach MS, Srichaikul K, Vidgen E, Mitchell S (2011). Nuts as a replacement for carbohydrates in the diabetic diet. Diabetes Care.

[81] Blanco Mejia S, Kendall CW, Viguiliouk E, Augustin LS, Ha V, Cozma AI (2014). Effect of tree nuts on metabolic syndrome criteria: a systematic review and meta-analysis of randomised controlled trials. BMJ Open.

[82] Holligan SD, West SG, Gebauer SK, Kay CD, Kris-Etherton PM (2014). A moderate-fat diet containing pistachios improves emerging markers of cardiometabolic syndrome in healthy adults with elevated LDL levels. Br J Nutr.

[83] Hernandez-Alonso P, Salas-Salvado J, Baldrich-Mora M, Mallol R, Correig X, Bullo M (2015). Effect of pistachio consumption on plasma lipoprotein subclasses in pre-diabetic subjects. Nutr Metab Cardiovasc Dis.

[84] Marventano S, Kolacz P, Castellano S, Galvano F, Buscemi S, Mistretta A (2015). A review of recent evidence in human studies of n-3 and n-6 PUFA intake on cardiovascular disease, cancer, and depressive disorders: does the ratio really matter?. Int J Food Sci Nutr.

[85] Oleszczak B, Szablewski L, Pliszka M (2012). The effect of hyperglycemia and hypoglycemia on glucose transport and expression of glucose transporters in human lymphocytes B and T: an in vitro study. Diabetes Res Clin Pract.

[86] Jaceldo-Siegl K, Sabate J, Rajaram S, Fraser GE (2004). Long-term almond supplementation without advice on food replacement induces favourable nutrient modifications to the habitual diets of free-living individuals. Br J Nutr.

[87] Ellis PR, Kendall CW, Ren Y, Parker C, Pacy JF, Waldron KW (2004). Role of cell walls in the bioaccessibility of lipids in almond seeds. Am J Clin Nutr.

[88] Novotny JA, Gebauer SK, Baer DJ (2012). Discrepancy between the Atwater factor predicted and empirically measured energy values of almonds in human diets. Am J Clin Nutr.

[89] Baer DJ, Gebauer SK, Novotny JA (2012). Measured energy value of pistachios in the human diet. Br J Nutr.

[90] Whelton PK (2014). Sodium, potassium, blood pressure, and cardiovascular disease in humans. Curr Hypertens Rep.

[91] Lai YH, Leu HB, Yeh WT, Chang HY, Pan WH. Low-normal serum potassium is associated with an increased risk of cardiovascular and all-cause death in community-based elderly. J Formos Med Assoc.2015;114(6):517–25. 10.1016/j.jfma.2015.01.001 26009484

[92] Farajian P, Katsagani M, Zampelas A (2010). Short-term effects of a snack including dried prunes on energy intake and satiety in normal-weight individuals. Eat Behav.

[93] Chang SKAC, Shahidi F (2016). Review of dried fruits: Phytochemicals, antioxidant efficacies, and health benefits. J Funct Foods.

[94] Karakaya S, El SN, Tas AA (2001). Antioxidant activity of some foods containing phenolic compounds. Int J Food Sci Nutr.

[95] Wu X, Beecher GR, Holden JM, Haytowitz DB, Gebhardt SE, Prior RL (2004). Lipophilic and hydrophilic antioxidant capacities of common foods in the United States. J Agric Food Chem.

[96] Zanotti I, Dall’Asta M, Mena P, Mele L, Bruni R, Ray S (2015). Atheroprotective effects of (poly)phenols: a focus on cell cholesterol metabolism. Food Funct.

